# Estimation of Vitamin K Content and Its Sources in the Diet of the Polish Participants of the PURE Study

**DOI:** 10.3390/nu14091917

**Published:** 2022-05-03

**Authors:** Bożena Regulska-Ilow, Dorota Różańska, Katarzyna Zatońska, Andrzej Szuba

**Affiliations:** 1 Department of Dietetics, Wroclaw Medical University, 50-556 Wroclaw, Poland; bozena.regulska-ilow@umw.edu.pl; 2 Department of Population Health, Wroclaw Medical University, 50-368 Wroclaw, Poland; katarzyna.zatonska@umw.edu.pl; 3 Department of Angiology, Hypertension and Diabetology, Wroclaw Medical University, 50-556 Wroclaw, Poland; andrzej.szuba@umw.edu.pl

**Keywords:** vitamin K, menaquinones, phylloquinone, dietary patterns, diet

## Abstract

The aim of the study was to estimate the content of K1, K2 (MK-n) as well as total K vitamins and their sources in the diets of 1985 PURE Poland study participants based on the FFQ questionnaire. Due to the pleiotropic effect of K vitamins, it is important to know their food sources depending on different eating habits. Total vitamin K in the diets amounted to 331.1 ± 151.5 µg/d and 358.6 ± 181.0 µg/d for men and women, respectively. Dietary patterns (DPs) were identified in the study group, and the relationship between them and vitamin K intake was assessed. The proportion of dominant products as sources of vitamin K in the fourth quartile of each of the three identified DPs did not change significantly compared to the proportion of these products as sources of vitamin K in all subjects. In the fourth quartile of individual DPs, vitamin K1 came mainly from vegetables (56.5–76.8%); K2 mainly from processed meat and high-fat cheese and cream (70.1–77.6%); and total K mainly from vegetables and processed meat (57.6–67.8%). Intakes of K vitamins were high and similar in terms of predominant vitamin K provider products, in groups of subjects whose diets were most consistent with the particular DP. In the absence of global findings on the most appropriate dietary content of MK-n vitamins other than phylloquinone, the estimated high content of these vitamins in the diets of the subjects compared with other nations suggests that their level in diets was satisfactory. Future studies should aim to determine the need for MK-n vitamins in terms of fulfilling all their functions in the body.

## 1. Introduction

Fat-soluble vitamin K has pleiotropic effects. Its main function is the modification of proteins to enable calcium-binding properties. Found in foods of plant origin as phylloquinone, vitamin K1 has antihemorrhagic properties and is used in the liver, where it activates calcium-binding proteins involved in clotting processes. A group of compounds called menaquinones (vitamin K2, MK-n, where n stands for the number of isoprenoid units from 4 to 13 forming the side chain) is found in foods of animal origin, with MK-4 and MK-7 forms thought to be the most important. Menaquinones are also synthesized by intestinal bacteria. Vitamin K2 activates matrix GLA protein (MGP), which binds calcium, and acts as an inhibitor of vascular calcification. This process strengthens the bones on the one hand, and prevents atherosclerotic remodeling of the arteries on the other [1]. Recently, vitamin K-dependent Gla-rich protein (GRP) has also been discovered and proposed to have an important role in vascular and valvular calcification inhibition [2]. A Nurses’ Health Study on the correlation between daily vitamin K intake and bone fragility indicated a 30% reduction in the risk of femoral neck fractures in women with daily vitamin K intake ≥ 109 μg, compared with women consuming < 109 μg [3]. Even though a diet very rich in vitamin K1 has no effect on the chronic complications of atherosclerosis [4], more than 90% of vitamin K in tissues is MK-4, which is most likely converted from vitamin K1 [5]. MK-4 is synthesized by the body from its precursor—phylloquinone—by the enzyme UBIAD1 [6,7]. Moreover, based on the study conducted by Ellis et al. [8], not only phylloquinone but also MK-4, MK-7 and MK-9 are all precursors to tissue MK-4, which was observed in mice. This is related to the greater availability of vitamin K2 in tissues, whereas vitamin K1 accumulates mainly in the liver [9]. Synthetically derived, water-soluble menadione (vitamin K3) is also one of the compounds that exhibit vitamin K biological activity. It is believed that it can be formed in the body and, as an intermediate product of metabolism, participates in the conversion of phylloquinone to MK-4 [10].

Vitamin K deficiency results in the formation of biologically inactive proteins in the body, i.e., proteins that lack carboxylated glutamic acid residues [11].

Vitamin K-dependent proteins (VKDP) play a key role in the pathogenesis of diseases such as: cardiovascular diseases, osteoporosis, and osteoarthritis. Recently, a new effect of vitamin K has been demonstrated, regardless of its role in the carboxylation of VKDP. It has been shown to be anti-inflammatory by inhibiting nuclear factor κB (NF-κB) signal transduction and to be protective against oxidative stress by blocking the production of reactive oxygen species [12]. Recent studies suggest that cellular redox status may play a key role in the regulation of different functions in the immune system [13].

The roles of the individual K-family vitamins are so diverse that many researchers believe that they should be classified as separate nutrients. Similarly, numerous functions of this group of vitamins have recently been described in the literature [1], particularly MK-4 functions related to tissue-specific conversion: participation in apoptosis and cell cycle induction in tumors and malignant cell lines [14,15,16]. The prospective, cohort PREDIMED study showed that subjects who increased their dietary supply of phylloquinone or menaquinones during the follow-up period compared with subjects who decreased or did not change their dietary supply of these components had a lower risk of cancer death and death overall. A significant relationship was also shown for dietary supply of phylloquinone, but not menaquinones, as well as risk of cardiovascular death [17]. A study in Norway conducted by Haugsgjerd et al. [18] also showed an association between higher dietary supply of vitamin K2 and reduced risk of incident ischemic heart disease, but no such relation was found for vitamin K1. A meta-analysis including 13 controlled clinical trials and 14 longitudinal studies showed that vitamin K supplementation significantly reduced vascular calcification [19]. This is important for the prevention of cardiovascular disease as up to more than 90% of atherosclerotic plaques are calcified, which in turn is associated with the degree of coronary stenosis [20]. In a study conducted by Dam et al. [21], a lower incidence of metabolic syndrome was observed with an increase in dietary supply of menaquinones.

Researchers also report significant effects of Gas6 protein activated by vitamin K2 on blood glucose levels in type 2 diabetes and pre-diabetic conditions [22,23].

Furthermore, it has been shown that MK-4 can mitigate inflammation and neutralize free radicals in the nervous system [24]. Vitamin K has also been observed to affect proteins present in the brain that have a protective effect on neurons. A correlation has also been shown between vitamin K levels and the function of enzymes involved in the biosynthesis of sphingolipids, a component of myelin and cell membranes [25].

A review paper published in 2020 by Palmer et al. [26] noted that few studies have assessed the vitamin K content of Western diets. Vitamin K content was assessed in different populations characterized by various dietary habits, i.e., the Netherlands [4], Spain [17], Norway [18], Germany [27], and Japan [28]. The content of vitamin K in the diet of Poles has not been studied as its content in food is not reported in the Polish Food Composition and Nutritional Value Tables [29]. The latest Norms of nutrition for the Polish population of 2020 reported that a literature review from the period of 2017–2020 showed a lack of studies on the intake and nutritional status of vitamin K in the Polish population [30].

Menaquinones are found in meat and liver, eggs (mainly MK-4), cheese and fermented dairy products (mainly MK-9) and pickled vegetables (mainly MK-7) [31]. Most of them are synthesized by bacteria capable of fermenting foods, intestinal bacteria and colon microbiota anaerobic bacteria [32]. The exception is MK-4, which is not a product of bacterial synthesis but of metabolic changes in phylloquinone in the intestinal mucosa and other organs of the human body [33].

Phylloquinone is found in dark green leafy vegetables (lettuce, broccoli, spinach, parsley, brassica), oils, soft margarines, spices (thyme, basil, coriander) and bread [1,26,34].

According to the available data, the dietary intake of vitamin K has not been assessed in Poland so far, so the estimation of its content will provide the first information on this subject. Given the importance of this vitamin due to its multidirectional effects, it is important to know its sources in the average diet. This will make it possible to indicate which products should be present in the daily diet to enrich it with this nutrient, and which products should be limited due to the possibility of interaction with drugs. Additional comparisons of dietary vitamin K content in relation to socio-demographic factors will allow us to indicate which groups may be at risk of inadequate vitamin K supply.

This study aims to estimate the content of vitamin K and its sources, before and after separation of dietary patterns, in the typical diet of Lower Silesian participants—urban and rural residents, both male and female—from the PURE Poland study.

## 2. Materials and Methods

### 2.1. Study Design and Participants 

The PURE Poland study is the Polish arm of the global, epidemiological, cohort-based Prospective Urban Rural Epidemiology study (PURE), involving over 150,000 adults from 17 low-, middle-, and high-income countries. The study objectives and description have been presented in previous papers [35,36].

The study group consisted of 2026 subjects, both male and female, from the city of Wroclaw and surrounding villages in Lower Silesia, who were recruited for the study by press and radio advertisements in the years 2007–2009. Enrollment in the study was voluntary and written informed consent was obtained from the participants for all study procedures. The study was approved by the Ethics Committee of Wroclaw Medical University (no. KB-443/2006). Inclusion criteria were: 35–70 years of age, energy value of daily rations in the range of 500–4000 kcal, and complete data on the study variables. A total of 1985 subjects were eventually included in the study. The characteristics of the study group are presented in Appendix A (Table A1). Table A1 shows the number and percentage of study participants in groups depending on age, place of residence, level of education, smoking status and BMI.

### 2.2. Dietary Assessment

The assessment of food intake, which served as the basis for the estimation of dietary intake of vitamin K, was performed using the standardized Food Frequency Questionnaire (FFQ). It was developed and validated within the PURE project for the study population from Lower Silesia [37]. It includes questions about the consumption of 154 food groups: milk and dairy products (20 products), fruits (13 products), vegetables (33 products), meat and eggs (23 products), breads and cereals (9 products), mixed foods (21 products), drinks (17 products), snacks (18 products) and 7 types of oils. The respondent’s task was to answer how often during the year preceding the survey they consumed particular products. The frequency of product consumption was defined in terms of month (never or less than once a month, 1–3 times a month), week (once, 2–4 times, 5–6 times a week) or day (once, 2–3 times, 4–5 times, 6 or more times a day). The frequency of consumption of each product was determined in relation to the average serving size reported in the questionnaire, expressed in household measures such as: 1 glass, 1 teaspoon, 1 plate or plateful, 1 slice, 1 medium fruit.

Based on the collected data, the intake of vitamins K1, K2 and total K with the whole daily ration and per 1000 kcal diet was estimated. Its main sources in the diet of urban and rural inhabitants of Lower Silesia were determined. The vitamin K content in the diet was calculated based on the United States Department of Agriculture (USDA) database [38], as well as available published data [19,31,39,40,41,42,43,44]. Vitamin K intake was assessed in relation to socio-demographic factors in the study group. Comparisons of dietary vitamin K content were made according to gender, place of residence, education level, smoking status, age and BMI. Based on the data obtained from the FFQ, dietary patterns were also extracted and the relationship between these patterns and vitamin K intake was evaluated.

### 2.3. Identification of Dietary Patterns

To identify dietary patterns in the study, foods from the FFQ were categorized into 25 groups based on their similar composition and nutritional value: milk and low-fat dairy products, high-fat cheese and cream, French fries, potatoes, eggs, red meat, processed meat, low-fat poultry, high-fat poultry, fish, unrefined cereals, refined cereals, mixed foods, soups, alcohol, sweets, beverages, sugar and honey, nuts and seeds and raisins, fruits, juices, vegetables, tea and coffee, animal fats, margarines and mayonnaise. Dishes characteristic of Polish cuisine, such as beans baked with meat in tomato sauce, cabbage leaves stuffed with meat and rice in tomato sauce, dumplings with meat, sauerkraut with sausage and meat (stewed), dumplings with potato and cottage cheese, and cooked vegetable salad with mayonnaise, were all classified as “mixed dishes”.

Principal components analysis (PCA) with varimax rotation was used to derive dietary patterns for the study group. Analysis was based on the frequency of daily intake of 25 food groups. Factor loadings ≥ 0.3 were used as cut-off points. Dietary patterns were named based on the characteristics of the main food groups. For each dietary pattern, the study group was divided into quartiles Q, based on the factor scores of their diets. Quartile 1 represented low adherence to each dietary pattern, while quartile 4 represented high adherence.

### 2.4. Statistical Analysis

All statistical analyses were conducted using the Statistica data analysis software system (version 13.3, TIBCO Software Inc. Palo Alto, Santa Clara, CA, USA). Differences in vitamin K intake between two groups were calculated using the Mann–Whitney U test. The comparison between socio-demographic factors and quartiles of vitamin K intake was assessed using Pearson’s chi-squared test. Correlation between linear variables (factor scores for each dietary pattern and daily vitamin K intake) was calculated using Pearson’s correlation coefficient. The values of the Pearson’s coefficient in the ranges from −1 to −0.5 and from 0.5 to 1.0 indicated a strong linear correlation between the examined variables, while the values in the ranges from −0.5 to 0.0 and from 0.0 to 0.5 indicated a poor correlation. The level of statistical significance for all analyses was set at *α* = 0.05.

## 3. Results

The content of vitamin K1, K2 and total K in the diets of 1985 subjects (733 men and 1252 women) was estimated and their sources in the diets of the studied subjects were indicated. The results are presented in Table 1; Table 2. Among 25 groups of food products (distinguished based on similar composition and nutritional value and degree of processing), vegetables, processed meat products, high-fat cheese and cream, and soups contributed the most to the total vitamin K in the diets of both genders. These provided 71% of the total vitamin K in men’s diets and 72% in women’s diets. In both groups, vitamin K1 came mainly from vegetables and significantly smaller amounts came from soups, margarines, and mayonnaise. These products provided 84% of the vitamin K1 in male diets and 80% in female diets. In both groups, the most significant sources of vitamin K2 were processed meat products and high-fat cheese and cream and, in smaller amounts, red meat, milk and low-fat dairy products. These products contributed 87% of vitamin K2 in male diets and 86% in female diets. Total vitamin K intake was estimated to be 331.1 ± 151.5 µg in men and 358.6 ± 181.0 µg in women.

Table 3 shows a comparison of dietary content of vitamin K1, K2, and total K per 1000 kcal by gender and place of residence. There was a significantly higher content of vitamin K1 and total K in the diets of women than men. Conversely, the opposite was observed for vitamin K2. Significantly higher content of vitamin K1 was found in the diets of urban inhabitants compared to rural dwellers; the opposite was shown for vitamin K2. A higher content of total vitamin K was estimated in the diets of urban women compared to rural women and in the diets of rural men compared to urban men.

Table 4 presents three dietary patterns characteristic of the studied group, identified with statistical methods on the basis of FFQ interview data, which were assigned factor loading values for 25 groups of food products and sources of vitamin K. Guided by knowledge about the importance of different groups of products for maintaining health, the dietary patterns were assigned the following names: unhealthy, healthy and traditional.

Table 5 shows quartiles of the identified dietary patterns, vitamin K intake in each pattern and the results of the Pearson’s correlation between the factor scores for each dietary pattern with vitamin K intake. A strong positive correlation was observed between the factor scores for the healthy dietary pattern and the vitamin K1 content of the diets of the study group and between the factor scores for the unhealthy dietary pattern and the vitamin K2 content of the diets. A strong positive correlation was also observed between the factor scores for the traditional dietary pattern and the dietary intake of vitamin K2 and total K.

Table 6, Table 7 and Table 8 show the intakes of vitamin K1, K2, and total K from each food group for individuals in the fourth quartile of the three identified dietary patterns. The major sources of vitamin K in each dietary pattern referred to products that provided more than 10% phylloquinone and menaquinones. In the fourth quartile of the unhealthy pattern, the predominant products provided 56% of vitamin K1 (vegetables), 70.1% of vitamin K2 (high-fat cheeses and processed meats), and 64.7% of total vitamin K (high-fat cheese, processed meats, and vegetables). In the fourth quartile of the healthy pattern, the predominant foods provided 71.1% of vitamin K1 (vegetables), 77.6% of vitamin K2 (low-fat dairy products, high-fat cheese and cream, and processed meat products), and 67.8% of total vitamin K (high-fat cheese, cream, processed meat products, and vegetables). In the fourth quartile of the traditional pattern, the predominant products provided 76.8% of vitamin K1 (vegetables and soups), 76.1% of vitamin K2 (high-fat cheese, cream, red and processed meat), and 57.6% of total vitamin K (vegetables and processed meat).

Table 9 shows the quartiles of vitamin K intake in relation to socio-demographic and anthropometric factors and smoking. There were no differences in quartiles of vitamin K1 and total K intake between subjects with BMI <24.9 to ≥ 30.0 kg/m2, as well as for total K with smoking status. Vitamin K2 intake in quartiles 1 to 4 was not associated with gender or age. As the vitamin K1 intake increased in quartiles 1 to 4, the number of women, urban inhabitants as well as people with secondary and higher education increased. In contrast, the number of men, rural residents, people with no/primary or vocational education, active smokers, and people over 65 years of age decreased. As the vitamin K2 content increased in quartiles 1 to 4, the number of subjects from rural areas, persons with no/primary or vocational education, active smokers, and subjects with BMI ≥ 30.0 kg/m2 increased. In contrast, the number of urban residents, those with higher education, non-smokers, and those with BMI <24.9 kg/m2 decreased. As total vitamin K increased in quartiles 1 to 4, the number of subjects aged 45 to 54 years increased, whereas the number of men and subjects over 65 years of age decreased.

## 4. Discussion

Considering the pleiotropic role played by vitamin K in the activation of proteins that perform very different functions in the body, it seems necessary to assess the content of this component in the diet, as well as its main sources in whole daily food rations. This seems even more important, as the most frequently discussed issue concerning vitamin K related to its content in the diet includes interactions of vitamin K with anticoagulants, coumarol derivatives (warfarin) used in cardiac patients, persons with liver diseases or with excessive hypermetabolism. Therapy with coumarol derivatives requires updating the drug dosage adjusted to, among other things, current vitamin K intake. A diet rich in phylloquinone, primarily containing large amounts of green leafy vegetables, attenuates the effects of vitamin K antagonist drugs [45,46]. As an alternative for users of coumarol derivatives, there may be foods that are not important sources of vitamin K and will not compromise the stability of their oral anticoagulant therapy. These include nuts and fruits, except for pine and cashew nuts, which, respectively, contain high levels of 53.9 and 34.8 µg of phylloquinone per 100 g of nut, as well as fruits with the exception of some berries (blueberries, blackberries), green fruits (kiwi, grapefruit) and dried plums and figs [47]. In contrast, the elderly and those with gastrointestinal diseases have an increased need for vitamin K. Many chronically used drugs, such as antidepressants, aspirin or some broad-spectrum antibiotics, change vitamin K metabolism, which also requires careful selection of its dose and sources in the diet [48].

The demand for vitamin K for the general population was established based on studies on the influence of vitamin K1 on the coagulation process. In the USA and Canada, the recommended daily doses are 120 µg for men and 90 µg for women [49]. In Great Britain, this is 1 µg/kg body weight/day [50]. Based on population data from European countries, the EFSA NDA Panel (Panel on Dietetic Products, Nutrition and Allergies) proposed reference values for vitamin K at the level of 70 μg/day for both women and men over 19 years of age [51]. These amounts may not be sufficient. In 2020, the Polish Team for Dietary Supplements of the Sanitary and Epidemiological Council determined the maximum dose of vitamin K in the recommended daily portion of the dietary supplement for adults at 200 μg. In Poland, the norm at the level of adequate intake (AI) for vitamin K was set only regarding phylloquinone and, for women over 16 years of age, it was set at 55 μg of phylloquinone/person/day, and 65 μg of phylloquinone/person/day for men over 16 years of age [30]. In this context, phylloquinone content in the diets of women and men estimated in the study herein—respectively, 205.2 ± 138.2 and 164.5 ± 94.9 µg/d—is four times higher in women and three times higher in men than the Polish norms.

It is still unclear how much vitamin K2 should be consumed for it to be most effective, especially since the bioavailability of vitamins from MK-4 to MK-13 vary. The authors investigated the bioavailability of the two most relevant components of vitamin K2, i.e., MK-4 and MK-7 provided as a breakfast supplement. MK-7 was well absorbed and reached maximum serum levels 6 h after intake and was detectable up to 48 h after intake. MK-4 was not detectable in the serum of all individuals at any time. Continuation of MK-4 supplementation did not increase serum MK-4 concentrations. However, subsequent administration of MK-7 significantly increased serum MK-7 concentrations in all subjects. Thus, it appears that MK-4 present in food does not affect vitamin K status as measured by serum vitamin K concentrations. In addition, it was shown that administering the same amount of vitamin K1 and MK-7 resulted in 10 times higher serum MK-7 concentrations than vitamin K1. It also seems that MK-7 (from diet or supplements) significantly increasing serum MK-7 concentrations may be of particular importance to non-hepatic tissues [44,52].

All types of vitamin K2 (except MK-4) can be produced by resident bacteria for their own respiratory cycle. The synthesis of different MK-n is strain-dependent. Minor differences in the structure of menaquinones determine different absorption, distribution in the body, metabolism, and excretion. Conversion of vitamin K1 to vitamin MK-4 in tissues and high concentration of MK-4, despite the supply of only vitamin K1, have been proven, suggesting that the body itself obtains menaquinones at low supply. The tissue-specific localization of MK-4 in extrahepatic tissues and the metabolic pathway for its synthesis from phylloquinone strongly suggest that there is an as-yet undiscovered, unique role for MK-4 that is independent of the currently recognized function of the coenzyme [50].

Therefore, the interpretation of the results of vitamin K intake from different sources does not allow a conclusion to be drawn on whether particular components of total vitamin K are present in the diet of the studied population in sufficient amounts to perform all functions of this vitamin, not only as phylloquinone in coagulation processes. Additionally, the possibility of comparing the results obtained in our study with the results of vitamin K intake in other populations is limited because there are no complete tables of product composition. In analyses conducted for the Dutch, German, and British populations, it was found that residents, on average, consume 30–50 µg/d of long-chain menaquinones, which represents approximately 25% of total vitamin K intake [1]. One hypothesis related to vitamin K2 intake assumes that the high intake of cheese in France and Mediterranean countries may possibly be responsible for the lower incidence of ischemic heart disease [53].

In the authors’ own work, the main sources of vitamin K1 are vegetables and soups, and despite its high content with respect to standards, its absorption from the diet is low (5–15%), as phylloquinone is strongly bound to chloroplast membranes. The half-life of vitamin K1 is also short. However, vitamin K1 undergoes a cycle of oxidation and reduction reactions in the liver that support its self-renewal and it is reused up to several thousands of times, which explains its low daily requirement relative to other vitamins [9,10,50].

In contrast, menaquinones (K2) are absorbed virtually completely in the presence of bile salts and have a long biological half-life [50]. Their sources for men and women in the PURE Poland study population were mainly processed and red meat, high-fat cheese and cream, as well as low-fat dairy products; it was estimated to be 153.3 ± 88 and 166.6 ± 92 µg/d, respectively.

In Japan, the diets of young women contained 230 µg/d of total vitamin K, with 94% of study participants aged 18–29 years meeting the AI standard of 60 µg/d. The proportion of vitamin K1, MK-4 and MK-7 intake in the total vitamin K pool was 67.7%, 7.3% and 24.9%, respectively. Vitamin K1 was mainly derived from vegetables and algae, and MK-7 from legumes, including fermented soybean products [28].

A study carried out in the Netherlands showed that phylloquinone accounted for 90% of the ingested vitamin K, with an average menaquinone intake of 30.8 ± 18.0 μg/d in men and 27.0 ± 15.1 μg/d in women. The intake of MK-4 accounted for 7.7 ± 3.4 μg/d and 6.3 ± 2.8 μg/d, respectively, while MK-5 to MK-10 accounted for 23.1 ± 16.3 μg/d and 20.7 ± 13.8 μg/d, respectively. Of these, cheese (53%), dairy products (19%), and meat (17%) provided the largest amounts, and MK-9 was the most abundant among the multi-chain menaquinones [4]. In the United States, menaquinone is added to poultry feed, so MK-4 formed from menaquinone is found in poultry and pork products, which are the main sources of MK-4 in the diets of Americans. MK-4 is also found in small amounts in milk, cheese, and butter. In the Rotterdam study, the mean intake of MK-5 and MK-10 in the male group was 23.1 ± 16.3 μg/d and 20.7 ± 13.8 μg/d in the female group. In Heideberg, spinach, lettuce, broccoli, and Brussels sprouts were the sources of 42% of ingested phylloquinone among men aged 40–65 years. Meats and meat products were the source of 37% of the MK-4 consumed, and 85% of the higher MK-5–9 came from dairy products. The European Food Safety Authority reported that menaquinone intakes in the United Kingdom ranged from 36 μg/d in women, 43 μg/d in men, to 54 μg/d in male adolescents [1,27,31].

In conclusion of the PURE Poland study, total vitamin K came mainly from vegetables, processed meat and high-fat cheeses in the diets of the studied population. The proportion of dominant products as sources of vitamin K in the fourth quartile of each of the three identified dietary patterns specific to the study group did not change significantly compared to the proportion of these products as sources of vitamin K in the studied population. In the fourth quartile of each dietary pattern, vitamin K1 came mainly from vegetables (56.5–76.8%); K2 mainly from processed meat and high-fat cheese and cream (70.1–77.6%); and total K came mainly from vegetables and processed meat (57.6–67.8%). In Europe, the most important sources of menaquinones are cheeses, while in Asian cultures it is natto—a product of soybean fermentation involving Bacillus subtilis natto bacteria. It contains large amounts of MK-7—900 to 1000 μg/100 g—and small amounts of MK-8 and phylloquinone—84 μg/100 g and 35 μg/100 g, respectively [1,44].

In the PURE Poland study, the highest amounts of vitamin K1 (Table 4) were found in the diets of women, urban inhabitants and better educated individuals. Vitamin K2 was highest in the diets of rural residents, the poorly educated, and people aged 45–64. Total K was dominant in the diets of women, urban inhabitants and people aged 55–64.

## 5. Conclusions

The intake of the K group vitamins by the PURE Poland study participants was high and similar in terms of the predominant products providing vitamin K, in subjects whose diets were most consistent with particular dietary patterns.

In the absence of global findings on the most appropriate dietary content of vitamins MK-n other than phylloquinone, their estimated high content in the subjects’ diets compared with other nations suggests that their amounts are satisfactory.

Future studies should aim to determine the amounts of MK-n vitamins required to fulfil all their functions in the body.

## Figures and Tables

**Table 1:** Vitamin K1, K2 and total K intake (µg/day) and its sources in the study group of men (*n* = 733). This table shows the intake of each food group (g/day) in the study group of men (*n* = 733), content of vitamin K1, K2 and total K (µg/day) in these food groups, and the percentage of vitamin intake from each food group in the overall diet.

Food Group	Food Group Intake (g/Day)	Vitamin K1 Intake from Each Food Group (µg/Day)		Vitamin K2 Intake from Each Food Group (µg/Day)		Total Vitamin K Intake from Each Food Group (µg/Day)	
	Mean ± SD	Mean ± SD	%	Mean ± SD	%	Mean ± SD	%
Milk, low-fat dairy	204.5 ± 210.5	0.56 ± 0.59	0.3	15.66 ± 14.01	9.4	16.21 ± 14.4	4.9
High-fat cheese, cream	28.0 ± 21.9	0.56 ± 0.47	0.3	34.31 ± 33.31	20.6	34.87 ± 33.67	10.5
Chips	6.4 ± 9.4	4.13 ± 6.10	2.5	-	-	4.13 ± 6.10	1.2
Potatoes	87.4 ± 56.1	1.88 ± 1.19	1.1	-	-	1.88 ± 1.19	0.6
Eggs	17.1 ± 17.4	0.70 ± 0.71	0.4	0.07 ± 0.07	0.0	0.78 ± 0.79	0.2
Red meat	30.1 ± 19.1	1.62 ± 1.15	1.0	17.38 ± 11.71	10.4	19.00 ± 12.76	5.7
Processed meat	48.8 ± 35.3	0.30 ± 0.31	0.2	78.05 ± 61.76	46.8	78.35 ± 61.96	23.7
Low-fat poultry	8.8 ± 10.2	0.01 ± 0.01	0.0	0.96 ± 1.12	0.6	0.97 ± 1.13	0.3
High-fat poultry	43.3 ± 33.3	5.08 ± 4.35	3.1	7.56 ± 6.43	4.5	12.64 ± 9.61	3.8
Fish	14.1 ± 11.5	0.11 ± 0.10	0.1	0.03 ± 0.02	0.0	0.13 ± 0.11	0.0
Unrefined grains	82.8 ± 87.5	0.71 ± 0.68	0.4	0.23 ± 0.62	0.1	0.94 ± 1.17	0.3
Refined grains	91.8 ± 69.6	4.23 ± 4.62	2.6	-	-	4.23 ± 4.62	1.3
Mixed dishes	35.0 ± 23.6	6.07 ± 4.34	3.7	3.06 ± 2.24	1.8	9.13 ± 6.34	2.8
Soups	252.3 ± 148.2	18.86 ± 15.03	11.5	3.30 ± 3.01	2.0	22.15 ± 16.37	6.7
Alcohol	111.9 ± 179.4	0.04 ± 0.09	0.0	-	-	0.04 ± 0.09	0.0
Sweets	46.3 ± 35.5	2.55 ± 2.07	1.6	0.16 ± 0.27	0.1	2.71 ± 2.14	0.8
Beverages	203.6 ± 341.9	0.25 ± 0.56	0.2	-	-	0.25 ± 0.56	0.1
Sugar and honey	18.6 ± 16.9	-	-	-	-	-	-
Nuts, seeds, raisins	12.4 ± 19.5	1.10 ± 1.89	0.7	-	-	1.10 ± 1.89	0.3
Fruits	246.9 ± 185.5	6.16 ± 5.54	3.7	-	-	6.16 ± 5.54	1.9
Juices	125.7 ± 132.8	1.71 ± 4.24	1.0	-	-	1.71 ± 4.24	0.5
Vegetables	269.6 ± 154.0	96.88 ± 83.39	58.9	2.00 ± 2.00	1.2	98.87 ± 84.17	29.9
Tea and coffee	878.4 ± 465.3	0.30 ± 0.27	0.2	-	-	0.30 ± 0.27	0.1
Animal fats	13.4 ± 15.5	0.56 ± 0.69	0.3	3.78 ± 4.18	2.3	4.35 ± 4.77	1.3
Margarines and mayonnaises	6.9 ± 6.9	10.10 ± 10.19	6.1	0.05 ± 0.09	0.0	10.15 ± 10.26	3.1
Diet overall	-	164.5 ± 94.9	100	166.6 ± 92.0	100	331.1 ± 151.5	100

SD—standard deviation.

**Table 2:** Vitamin K1, K2 and total K intake (µg/day) and its sources in the study group of women (*n* = 1252). This table shows the intake of each food group (g/day) in the study group of women (*n* = 1252), content of vitamin K1, K2 and total K (µg/day) in these food groups, and the percentage of vitamin intake from each food group in the overall diet.

Food Group	Food Group Intake (g/Day)	Vitamin K1 Intake from Each Food Group (µg/Day)		Vitamin K2 Intake from Each Food Group (µg/Day)		Total Vitamin K Intake from Each Food Group (µg/Day)	
	Mean ± SD	Mean ± SD	%	Mean ± SD	%	Mean ± SD	%
Milk, low-fat dairy	234.9 ± 225.4	0.63 ± 0.63	0.3	19.93 ± 15.23	13.0	20.56 ± 15.61	5.7
High-fat cheese, cream	29.0 ± 23.3	0.57 ± 0.49	0.3	37.17 ± 37.26	24.2	37.74 ± 37.66	10.5
Chips	5.4 ± 9.0	3.52 ± 5.80	1.7	-	-	3.52 ± 5.80	1.0
Potatoes	82.4 ± 58.9	1.77 ± 1.25	0.9	-	-	1.77 ± 1.25	0.5
Eggs	14.6 ± 13.5	0.60 ± 0.55	0.3	0.06 ± 0.06	0.0	0.66 ± 0.61	0.2
Red meat	24.7 ± 16.6	1.38 ± 1.00	0.7	14.29 ± 10.04	9.3	15.67 ± 10.96	4.4
Processed meat	39.4 ± 30.9	0.24 ± 0.28	0.1	59.97 ± 52.51	39.1	60.21 ± 52.70	16.8
Low-fat poultry	10.9 ± 11.9	0.01 ± 0.02	0.0	1.18 ± 1.30	0.8	1.19 ± 1.31	0.3
High-fat poultry	48.1 ± 36.3	5.21 ± 4.56	2.5	8.29 ± 6.73	5.4	13.49 ± 10.01	3.8
Fish	13.7 ± 10.8	0.10 ± 0.10	0.1	0.03 ± 0.02	0.0	0.13 ± 0.11	0.0
Unrefined grains	92.3 ± 85.3	0.78 ± 0.64	0.4	0.29 ± 0.65	0.2	1.08 ± 1.16	0.3
Refined grains	68.7 ± 60.1	2.80 ± 3.48	1.4	-	-	2.80 ± 3.48	0.8
Mixed dishes	32.2 ± 21.5	5.66 ± 4.06	2.8	2.93 ± 2.12	1.9	8.60 ± 5.90	2.4
Soups	246.7 ± 141.7	18.39 ± 14.32	9.0	3.17 ± 2.95	2.1	21.56 ± 15.67	6.0
Alcohol	24.2 ± 59.7	0.03 ± 0.07	0.0	-	-	0.03 ± 0.07	-
Sweets	49.2 ± 38.8	2.75 ± 2.36	1.3	0.16 ± 0.26	0.1	2.91 ± 2.41	0.8
Beverages	198.8 ± 344.5	0.27 ± 0.57	0.1	-	-	0.27 ± 0.57	0.1
Sugar and honey	15.8 ± 15.5	-	-	-	-	-	-
Nuts, seeds, raisins	15.6 ± 23.9	1.32 ± 2.21	0.6	-	-	1.32 ± 2.1	0.4
Fruits	325.9 ± 219.2	9.12 ± 8.05	4.4	-	-	9.12 ± 8.05	2.5
Juices	138.0 ± 145.2	3.76 ± 6.88	1.8	-	-	3.76 ± 6.88	1.0
Vegetables	339.0 ± 212.8	135.98 ± 125.46	66.3	2.11 ± 2.02	1.4	138.10 ± 126.14	38.5
Tea and coffee	984.1 ± 467.3	0.34 ± 0.26	0.2	-	-	0.34 ± 0.26	0.1
Animal fats	14.1 ± 15.8	0.60 ± 0.70	0.3	3.76 ± 4.22	2.4	4.36 ± 4.83	1.2
Margarines and mayonnaises	6.5 ± 6.3	9.37 ± 9.30	4.6	0.04 ± 0.07	0.0	9.41 ± 9.35	2.6
Diet overall	-	205.2 ± 138.2	100	153.4 ± 87.7	100	358.6 ± 181.0	100

SD—standard deviation.

**Table 3:** Vitamin K1, K2 and total K intake (µg/1000 kcal) in the study population by gender and residence. This table shows the mean ± standard deviation, quartile 1, median and quartile 3 of vitamin K1, K2 and total K intake (µg/1000 kcal) in the whole study population and in the age and place of residence groups, and the results of Mann–Whitney U test comparison between groups.

Study Group	Mean ± SD	Quartile 1	Median	Quartile 3	*p* *
	Vitamin K1 (µg/1000 kcal)				
Total (*n* = 1985)	94.2 ± 58.9	61.7	81.9	108.1	
Men (*n* = 733)	79.6 ± 43.4	54.9	70.8	93.7	<0.0001
Women (*n* = 1252)	102.7 ± 64.9	66.4	88.0	117.8	
Men—urban (*n* = 453)	83.9 ± 50.7	55.7	75.5	100.5	0.0029
Men—rural (*n* = 280)	72.5 ± 26.3	54.8	67.1	84.5	
Women—urban (*n* = 739)	117.6 ± 76.2	74.9	102.9	137.0	<0.0001
Women—rural (*n* = 513)	81.2 ± 33.6	60.1	75.3	95.6	
	**Vitamin K2 (** **µg/1000 kcal)**				
Total (*n* = 1985)	75.0 ± 30.5	54.0	70.3	92.7	
Men (*n* = 733)	78.0 ± 32.5	55.2	72.3	95.6	0.0037
Women (*n* = 1252)	73.3 ± 29.1	53.1	68.6	90.2	
Men—urban (*n* = 453)	72.4 ± 33.3	51.1	66.5	87.2	<0.0001
Men—rural (*n* = 280)	87.0 ± 29.1	66.7	85.4	106.7	
Women—urban (*n* = 739)	66.6 ± 27.7	47.9	62.3	82.9	<0.0001
Women—rural (*n* = 513)	82.8 ± 28.4	63.2	78.1	103.2	
	**Total Vitamin K (** **µg/1000 kcal)**				
Total (*n* = 1985)	169.2 ± 63.7	132.3	160.1	192.2	
Men (*n* = 733)	157.6 ± 54.1	123.8	149.8	180.3	<0.0001
Women (*n* = 1252)	175.9 ± 67.8	137.0	165.4	198.1	
Men—urban (*n* = 453)	156.3 ± 62.5	117.8	145.3	179.7	0.0027
Men—rural (*n* = 280)	159.5 ± 36.8	134.9	155.2	181.2	
Women—urban (*n* = 739)	184.3 ± 80.2	137.8	168.4	208.3	<0.0001
Women—rural (*n* = 513)	163.9 ± 41.4	135.8	161.5	184.6	

SD—standard deviation; * Mann–Whitney U test.

**Table 4:** Factor loading matrix for the dietary patterns derived in the study group.* This table shows factor loading matrix derived by principal components analysis (PCA) with varimax rotation, where factor load ≥ 0.3 was used as the cut-off point. Dietary patterns were named based on the characteristics of the main food groups.

Food Group	Unhealthy Dietary Pattern	Healthy Dietary Pattern	Traditional Dietary Pattern
Milk, low-fat dairy	0.24	**0.45**	0.01
High-fat cheese, cream	**0.54**	0.27	0.10
Chips	**0.45**	−0.21	0.23
Potatoes	0.10	0.01	**0.33**
Eggs	**0.43**	−0.05	0.10
Red meat	0.25	−0.09	**0.68**
Processed meat	**0.54**	−0.17	**0.50**
Low-fat poultry	−0.20	0.28	**0.43**
High-fat poultry	**0.34**	0.12	**0.51**
Fish	0.20	0.14	**0.48**
Unrefined grains	−0.10	**0.51**	0.14
Refined grains	**0.57**	−0.27	0.20
Mixed dishes	0.13	0.14	**0.70**
Soups	0.01	0.19	**0.65**
Alcohol	−0.05	−0.14	0.21
Sweets	**0.53**	0.29	0.20
Beverages	0.01	0.13	−0.09
Sugar and honey	**0.49**	0.08	−0.05
Nuts, seeds, raisins	−0.01	**0.60**	0.05
Fruits	−0.03	**0.66**	0.10
Juices	**0.30**	0.29	0.19
Vegetables	−0.03	**0.61**	**0.42**
Tea and coffee	**0.33**	0.22	−0.16
Animal fats	**0.44**	0.06	0.03
Margarines and mayonnaises	**0.48**	−0.10	0.07

* Factor loadings ≥ 0.3 are bolded.

**Table 5:** Vitamin K1, K2 and total K intake (µg/day) across quartiles of dietary patterns derived in the study group. This table shows mean ± standard deviation of vitamin K1, K2 and total K intake (µg/day) across quartiles of three dietary patterns derived in the study group and Pearson correlation coefficient between factor scores for each dietary pattern and daily vitamin K intake.

Dietary Patterns	Q1	Q2	Q3	Q4	Pearson Correlation Coefficient (r) *
	Vitamin K1 (µg/Day)				
Unhealthy DP	201.7 ± 163.3	173.3 ± 110.0	189.5 ± 112.8	196.2 ± 105.9	0.01
Healthy DP	132.5 ± 59.5	154.7 ± 73.3	190.4 ± 87.5	282.9 ± 182.5	**0.51**
Traditional DP	145.2 ± 95.7	159.7 ± 83.6	185.8 ± 94.6	269.8 ± 169.3	**0.43**
	**Vitamin K2 (** **µg/Day)**				
Unhealthy DP	103.1 ± 63.4	129.8 ± 71.5	163.5 ± 75.3	236.5 ± 85.2	**0.58**
Healthy DP	157.9 ± 87.2	155.6 ± 83.1	152.1 ± 82.8	167.4 ± 103.0	**0.07**
Traditional DP	105.2 ± 60.9	140.3 ± 74.8	173.8 ± 80.8	213.7 ± 98.9	**0.52**
	**Total Vitamin K (** **µg/Day)**				
Unhealthy DP	304.7 ± 190.1	303.0 ± 152.0	353.0 ± 154.5	432.8 ± 151.9	**0.31**
Healthy DP	290.5 ± 125.2	310.2 ± 127.2	342.5 ± 127.1	450.3 ± 232.4	**0.41**
Traditional DP	250.4 ± 123.1	300.0 ± 115.1	359.6 ± 122.5	483.4 ± 208.5	**0.58**

DP—dietary pattern; * Q1/2/3/4—quartiles 1/2/3/4; correlation coefficients with *p* value > 0.05 are bolded.

**Table 6:** Vitamin K1, K2 and total K intake (µg/day) in the fourth quartile of the unhealthy dietary pattern derived from the study group. This table shows the intake of each food group (g/day) in subjects in the fourth quartile of unhealthy dietary pattern derived from the study group, content of vitamin K1, K2 and total K (µg/day) in this quartile, and the percentage of vitamin intake from each food group in the diet of subjects in this quartile.

Food Group	Unhealthy Dietary Pattern—4th Quartile (*n* = 497)						
	Food Group Intake (g/Day)	Vitamin K1 Intake from Each Food Group (µg/Day)		Vitamin K2 Intake from Each Food Group (µg/Day)		Total Vitamin K Intake from Each Food Group (µg/Day)	
	Mean ± SD	Mean ± SD	%	Mean ± SD	%	Mean ± SD	%
Milk, low-fat dairy	301.6 ± 269.2	0.81 ± 0.74	0.4	20.95 ± 15.30	8.9	21.76 ± 15.76	5.0
High-fat cheese, cream	46.3 ± 24.8	0.92 ± 0.54	0.5	60.10 ± 38.73	25.4	61.02 ± 39.11	14.1
Chips	11.8 ± 11.7	7.64 ± 7.57	3.9	0.00 ± 0.00	0.0	7.64 ± 7.57	1.8
Potatoes	88.0 ± 58.4	1.88 ± 1.23	1.0	0.00 ± 0.00	0.0	1.88 ± 1.23	0.4
Eggs	24.5 ± 20.7	1.00 ± 0.85	0.5	0.11 ± 0.09	0.0	1.11 ± 0.94	0.3
Red meat	32.9 ± 17.5	1.74 ± 1.05	0.9	20.08 ± 10.65	8.5	21.82 ± 11.56	5.0
Processed meat	70.3 ± 35.7	0.48 ± 0.36	0.2	105.73 ± 58.78	44.7	106.22 ± 58.98	24.5
Low-fat poultry	7.4 ± 10.6	0.01 ± 0.02	0.0	0.80 ± 1.16	0.3	0.81 ± 1.16	0.2
High-fat poultry	65.7 ± 39.0	6.08 ± 4.40	3.1	12.16 ± 7.37	5.1	18.24 ± 10.41	4.2
Fish	18.0 ± 9.8	0.14 ± 0.09	0.1	0.03 ± 0.02	0.0	0.17 ± 0.09	0.0
Unrefined grains	78.6 ± 67.3	0.62 ± 0.54	0.3	0.21 ± 0.48	0.1	0.84 ± 0.89	0.2
Refined grains	129.9 ± 66.4	6.22 ± 4.68	3.2	0.00 ± 0.00	0.0	6.22 ± 4.68	1.4
Mixed dishes	38.2 ± 19.4	6.47 ± 3.67	3.3	3.34 ± 2.03	1.4	9.81 ± 5.46	2.3
Soups	258.5 ± 111.5	17.22 ± 10.92	8.8	4.08 ± 2.88	1.7	21.30 ± 12.30	4.9
Alcohol	49.9 ± 104.3	0.03 ± 0.06	0.0	0.00 ± 0.00	0.0	0.03 ± 0.06	0.0
Sweets	76.2 ± 45.0	4.34 ± 2.72	2.2	0.17 ± 0.26	0.1	4.51 ± 2.79	1.0
Beverages	198.9 ± 329.8	0.48 ± 0.72	0.2	0.00 ± 0.00	0.0	0.48 ± 0.72	0.1
Sugar and honey	27.1 ± 18.3	0.00 ± 0.00	0.0	0.00 ± 0.00	0.0	0.00 ± 0.00	0.0
Nuts, seeds, raisins	13.6 ± 25.6	1.12 ± 2.31	0.6	0.00 ± 0.00	0.0	1.12 ± 2.31	0.3
Fruits	289.9 ± 203.5	6.78 ± 6.67	3.5	0.00 ± 0.00	0.0	6.78 ± 6.67	1.6
Juices	193.1 ± 156.6	3.81 ± 6.45	1.9	0.00 ± 0.00	0.0	3.81 ± 6.45	0.9
Vegetables	308.6 ± 174.7	111.03 ± 92.15	56.6	2.14 ± 1.72	0.9	113.17 ± 92.77	26.1
Tea and coffee	1127.0 ± 435.7	0.40 ± 0.28	0.2	0.00 ± 0.00	0.0	0.40 ± 0.28	0.1
Animal fats	23.3 ± 19.9	0.98 ± 0.90	0.5	6.56 ± 5.28	2.8	7.54 ± 6.03	1.7
Margarines and mayonnaises	11.0 ± 7.7	16.02 ± 11.60	8.2	0.07 ± 0.11	0.0	16.09 ± 11.68	3.7
Total	-	196.24 ± 105.87	100.0	236.53 ± 85.21	100.0	432.77 ± 151.87	100.0

SD—standard deviation.

**Table 7:** Vitamin K1, K2 and total K intake (µg/day) in the fourth quartile of healthy dietary pattern derived in the study group. This table shows the intake of each food group (g/day) in subjects in the fourth quartile of healthy dietary pattern derived from the study group, content of vitamin K1, K2 and total K (µg/day) in this quartile, and the percentage of vitamin intake from each food group in the diet of subjects in this quartile.

Food Group	Healthy Dietary Pattern—Quartile 4th (*n* = 497)						
	Food Group Intake (g/Day)	Vitamin K1 Intake from Each Food Group (µg/Day)		Vitamin K2 Intake from Each Food Group (µg/Day)		Total Vitamin K Intake from Each Food Group (µg/Day)	
	Mean ± SD	X ± SD	%	Mean ± SD	%	Mean ± SD	%
Milk, low-fat dairy	355.7 ± 293.2	0.95 ± 0.79	0.3	28.66 ± 18.51	17.1	29.61 ± 18.99	6.6
High-fat cheese, cream	35.3 ± 28.2	0.71 ± 0.60	0.3	47.17 ± 47.90	28.2	47.88 ± 48.38	10.6
Chips	3.5 ± 8.3	2.27 ± 5.34	0.8	0.00 ± 0.00	0.0	2.27 ± 5.34	0.5
Potatoes	83.7 ± 63.3	1.79 ± 1.34	0.6	0.00 ± 0.00	0.0	1.79 ± 1.34	0.4
Eggs	14.6 ± 13.3	0.60 ± 0.55	0.2	0.06 ± 0.06	0.0	0.66 ± 0.60	0.1
Red meat	24.5 ± 20.3	1.31 ± 1.21	0.5	13.29 ± 12.03	7.9	14.61 ± 13.18	3.2
Processed meat	35.5 ± 33.1	0.19 ± 0.27	0.1	54.06 ± 55.70	32.3	54.25 ± 55.90	12.0
Low-fat poultry	14.3 ± 14.0	0.02 ± 0.02	0.0	1.55 ± 1.52	0.9	1.56 ± 1.53	0.3
High-fat poultry	51.3 ± 43.2	5.56 ± 5.23	2.0	8.60 ± 7.83	5.1	14.16 ± 11.83	3.1
Fish	16.9 ± 14.3	0.13 ± 0.13	0.0	0.03 ± 0.03	0.0	0.16 ± 0.14	0.0
Unrefined grains	150.9 ± 122.1	1.25 ± 0.82	0.4	0.61 ± 1.04	0.4	1.86 ± 1.71	0.4
Refined grains	54.1 ± 55.0	1.80 ± 2.81	0.6	0.00 ± 0.00	0.0	1.80 ± 2.81	0.4
Mixed dishes	37.4 ± 29.1	6.72 ± 5.48	2.4	3.45 ± 2.81	2.1	10.17 ± 7.97	2.3
Soups	287.9 ± 184.3	22.98 ± 18.28	8.1	3.43 ± 3.61	2.0	26.41 ± 20.02	5.9
Alcohol	43.4 ± 85.0	0.05 ± 0.10	0.0	0.00 ± 0.00	0.0	0.05 ± 0.10	0.0
Sweets	60.5 ± 49.8	3.35 ± 3.03	1.2	0.20 ± 0.32	0.1	3.55 ± 3.10	0.8
Beverages	268.3 ± 407.3	0.28 ± 0.58	0.1	0.00 ± 0.00	0.0	0.28 ± 0.58	0.1
Sugar and honey	18.2 ± 18.2	0.00 ± 0.00	0.0	0.00 ± 0.00	0.0	0.00 ± 0.00	0.0
Nuts, seeds, raisins	31.9 ± 36.3	2.79 ± 3.46	1.0	0.00 ± 0.00	0.0	2.79 ± 3.46	0.6
Fruits	499.4 ± 254.0	14.35 ± 9.81	5.1	0.00 ± 0.00	0.0	14.35 ± 9.81	3.2
Juices	179.1 ± 190.0	5.40 ± 9.16	1.9	0.00 ± 0.00	0.0	5.40 ± 9.16	1.2
Vegetables	481.0 ± 256.2	201.03 ± 168.93	71.1	2.63 ± 2.70	1.6	203.66 ± 169.58	45.2
Tea and coffee	1052.8 ± 511.0	0.36 ± 0.28	0.1	0.00 ± 0.00	0.0	0.36 ± 0.28	0.1
Animal fats	13.9 ± 16.2	0.60 ± 0.72	0.2	3.61 ± 4.32	2.2	4.21 ± 4.94	0.9
Margarines and mayonnaises	5.7 ± 6.5	8.38 ± 9.60	3.0	0.03 ± 0.07	0.0	8.41 ± 9.65	1.9
Total	-	282.88 ± 182,49	100.0	167.38 ± 103.03	100.00	450.26 ± 232,39	100.0

SD—standard deviation.

**Table 8:** Vitamin K1, K2 and total K intake (µg/day) in the fourth quartile of traditional dietary pattern derived from the study group. This table shows the intake of each food group (g/day) in subjects in the fourth quartile of traditional dietary pattern derived from the study group, content of vitamin K1, K2 and total K (µg/day) in this quartile, and the percentage of vitamin intake from each food group in the diet of subjects in this quartile.

Food Group	Traditional Dietary Pattern—Quartile 4th (*n* = 497)						
	Food Group Intake (g/Day)	Vitamin K1 Intake from Each Food Group (µg/Day)		Vitamin K2 Intake from Each Food Group (µg/Day)		Total Vitamin K Intake from Each Food Group (µg/Day)	
	Mean ± SD	Mean ± SD	%	Mean ± SD	%	Mean ± SD	%
Milk, low-fat dairy	233.5 ± 229.9	0.63 ± 0.64	0.2	19.65 ± 16.68	9.2	20.28 ± 17.12	4.2
High-fat cheese, cream	32.1 ± 25.7	0.64 ± 0.55	0.2	41.65 ± 41.29	19.5	42.29 ± 41.72	8.7
Chips	8.3 ± 11.8	5.34 ± 7.65	2.0	0.00 ± 0.00	0.0	5.34 ± 7.65	1.1
Potatoes	112.7 ± 62.6	2.41 ± 1.32	0.9	0.00 ± 0.00	0.0	2.41 ± 1.32	0.5
Eggs	17.1 ± 16.9	0.70 ± 0.69	0.3	0.07 ± 0.07	0.0	0.77 ± 0.77	0.2
Red meat	42.5 ± 21.7	2.31 ± 1.37	0.9	23.60 ± 13.52	11.0	25.90 ± 14.78	5.4
Processed meat	61.3 ± 37.6	0.40 ± 0.35	0.1	97.45 ± 69.96	45.6	97.85 ± 70.20	20.2
Low-fat poultry	17.1 ± 15.4	0.02 ± 0.02	0.0	1.85 ± 1.69	0.9	1.87 ± 1.70	0.4
High-fat poultry	70.0 ± 44.1	7.88 ± 5.67	2.9	11.99 ± 8.23	5.6	19.87 ± 12.49	4.1
Fish	20.5 ± 14.6	0.15 ± 0.13	0.1	0.04 ± 0.03	0.0	0.19 ± 0.14	0.0
Unrefined grains	103.3 ± 86.2	0.89 ± 0.67	0.3	0.30 ± 0.61	0.1	1.19 ± 1.14	0.2
Refined grains	92.2 ± 73.2	3.30 ± 4.02	1.2	0.00 ± 0.00	0.0	3.30 ± 4.02	0.7
Mixed dishes	53.3 ± 29.5	9.48 ± 5.50	3.5	4.67 ± 3.02	2.2	14.15 ± 8.15	2.9
Soups	376.4 ± 176.6	29.65 ± 18.78	11.0	4.92 ± 3.90	2.3	34.58 ± 20.24	7.2
Alcohol	101.2 ± 189.8	0.04 ± 0.09	0.0	0.00 ± 0.00	0.0	0.04 ± 0.09	0.0
Sweets	58.9 ± 43.7	3.26 ± 2.59	1.2	0.18 ± 0.30	0.1	3.43 ± 2.66	0.7
Beverages	157.6 ± 290.8	0.32 ± 0.61	0.1	0.00 ± 0.00	0.0	0.32 ± 0.61	0.1
Sugar and honey	15.9 ± 15.9	0.00 ± 0.00	0.0	0.00 ± 0.00	0.0	0.00 ± 0.00	0.0
Nuts, seeds, raisins	17.4 ± 25.1	1.53 ± 2.42	0.6	0.00 ± 0.00	0.0	1.53 ± 2.42	0.3
Fruits	334.9 ± 231.1	8.97 ± 7.87	3.3	0.00 ± 0.00	0.0	8.97 ± 7.87	1.9
Juices	168.9 ± 176.6	3.35 ± 6.68	1.2	0.00 ± 0.00	0.0	3.35 ± 6.68	0.7
Vegetables	438.4 ± 253.2	177.55 ± 159.39	65.8	3.14 ± 2.68	1.5	180.68 ± 160.04	37.4
Tea and coffee	843.9 ± 500.7	0.30 ± 0.27	0.1	0.00 ± 0.00	0.0	0.30 ± 0.27	0.1
Animal fats	14.2 ± 16.3	0.59 ± 0.72	0.2	4.15 ± 4.67	1.9	4.74 ± 5.26	1.0
Margarines and mayonnaises	6.9 ± 6.5	10.03 ± 9.64	3.7	0.04 ± 0.08	0.0	10.07 ± 9.70	2.1
Total	-	269.75 ± 169.32	100.0	213.69 ± 98.94	100.0	483.45 ± 208.55	100.0

SD—standard deviation.

**Table 9:** Characteristics of the study group by quartiles of vitamin K1, K2 and total K intake (µg/1000 kcal). This table shows number of subjects in the study group across quartiles of vitamin K1, K2 and total K intake (µg/1000 kcal) divided by gender, place of residence, level of education, smoking status, age, BMI and the results of Pearson’s chi-squared test used for comparison between groups.

Variables		Q1	Q2	Q3	Q4	*p* *
		Vitamin K1 (µg/1000 kcal)				
Gender	Men *n* (%)	254 (51.2)	212 (42.7)	162 (32.7)	105 (21.1)	<0.0001
	Women *n* (%)	242 (48.8)	284 (57.3)	334 (67.3)	392 (78.9)	
Place of residence	Urban *n* (%)	257 (51.8)	238 (48.0)	289 (58.3)	408 (82.1)	<0.0001
	Rural *n* (%)	239 (48.2)	258 (52.0)	207 (41.7)	89 (17.9)	
Level of education	Non/primary *n* (%)	106 (21.4)	91 (18.4)	61 (12.3)	28 (5.6)	<0.0001
	Vocational *n* (%)	101 (20.4)	100 (20.2)	74 (14.9)	39 (7.9)	
	Secondary *n* (%)	178 (35.9)	191 (38.5)	204 (41.1)	213 (42.9)	
	Higher *n* (%)	111 (22.4)	114 (23.0)	157 (31.7)	217 (43.7)	
Smoking status	Never *n* (%)	239 (48.2)	239 (48.2)	212 (42.7)	245 (49.3)	0.0130
	Previous *n* (%)	136 (27.4)	161 (32.5)	165 (33.3)	168 (33.8)	
	Current *n* (%)	121 (24.2)	96 (19.4)	119 (24.0)	84 (16.9)	
Age (years old)	<45 *n* (%)	101 (20.4)	92 (18.6)	99 (20.0)	55 (11.1)	<0.0001
	45–54 *n* (%)	138 (27.8)	145 (29.2)	142 (28.6)	169 (34.0)	
	55–64 *n* (%)	153 (30.9)	190 (38.3)	187 (37.7)	199 (40.0)	
	≥65 *n* (%)	104 (21.0)	69 (13.9)	68 (13.7)	74 (14.9)	
BMI (kg/m^2^)	<24.9 *n* (%)	149 (30.0)	138 (27.8)	144 (29.0)	146 (29.4)	0.9824
	25–29.9 *n* (%)	191 (38.5)	204 (41.1)	202 (40.7)	197 (39.6)	
	≥30 *n* (%)	156 (31.5)	154 (31.1)	150 (30.2)	154 (31.0)	
		**Vitamin K2 (** **µg/1000 kcal)**				
Gender	Men *n* (%)	170 (34.3)	169 (34.1)	188 (37.9)	206 (41.5)	0.0503
	Women *n* (%)	326 (65.7)	327 (65.9)	308 (62.1)	291 (58.6)	
Place of residence	Urban *n* (%)	386 (77.8)	330 (66.5)	262 (52.8)	214 (43.1)	<0.0001
	Rural *n* (%)	110 (22.2)	166 (33.5)	234 (47.2)	283 (56.9)	
Level of education	Non/primary *n* (%)	51 (10.3)	48 (9.7)	71 (14.3)	116 (23.3)	<0.0001
	Vocational *n* (%)	46 (9.3)	75 (15.1)	98 (19.8)	95 (19.1)	
	Secondary *n* (%)	199 (40.1)	223 (45.0)	183 (36.9)	181 (36.4)	
	Higher *n* (%)	200 (40.3)	150 (30.2)	144 (29.0)	105 (21.1)	
Smoking status	Never *n* (%)	248 (50.0)	239 (48.2)	234 (47.2)	214 (43.1)	0.0235
	Previous *n* (%)	165 (33.3)	161 (32.5)	149 (30.0)	155 (31.2)	
	Current *n* (%)	83 (16.7)	96 (19.4)	113 (22.8)	128 (25.8)	
Age (years old)	<45 *n* (%)	72 (14.5)	96 (19.4)	97 (19.6)	82 (16.5)	0.1310
	45–54 *n* (%)	138 (27.8)	140 (28.2)	158 (31.9)	158 (31.8)	
	55–64 *n* (%)	193 (38.9)	186 (37.5)	175 (35.3)	175 (35.2)	
	≥65 *n* (%)	93 (18.8)	74 (14.9)	66 (13.3)	82 (16.5)	
BMI (kg/m^2^)	<24.9 *n* (%)	159 (32.1)	145 (29.2)	146 (29.4)	127 (25.6)	0.0437
	25–29.9 *n* (%)	209 (42.1)	203 (40.9)	186 (37.5)	196 (39.4)	
	≥30 *n* (%)	128 (25.8)	148 (29.8)	164 (33.1)	174 (35.0)	
		**Total Vitamin K (** **µg/1000 kcal)**				
Gender	Men *n* (%)	234 (47.2)	193 (38.9)	167 (33.7)	139 (28.0)	<0.0001
	Women *n* (%)	262 (52.8)	303 (61.1)	329 (66.3)	358 (72.0)	
Place of residence	Urban *n* (%)	326 (65.7)	265 (53.4)	258 (52.0)	343 (69.0)	<0.0001
	Rural *n* (%)	170 (34.3)	231 (46.6)	238 (48.0)	154 (31.0)	
Level of education	Non/primary *n* (%)	72 (14.5)	85 (17.1)	74 (14.9)	55 (11.1)	0.0007
	Vocational *n* (%)	74 (14.9)	83 (16.7)	100 (20.2)	57 (11.5)	
	Secondary *n* (%)	197 (39.7)	200 (40.3)	177 (35.7)	212 (42.7)	
	Higher *n* (%)	153 (30.9)	128 (25.8)	145 (29.2)	173 (34.8)	
Smoking status	Never *n* (%)	246 (49.6)	228 (46.0)	245 (49.4)	216 (43.5)	0.5071
	Previous *n* (%)	151 (30.4)	159 (32.1)	152 (30.7)	168 (33.8)	
	Current *n* (%)	99 (20.0)	109 (22.0)	99 (20.0)	113 (22.7)	
Age (years old)	<45 *n* (%)	90 (18.2)	111 (22.4)	90 (18.2)	56 (11.3)	0.0001
	45–54 *n* (%)	132 (26.6)	137 (27.6)	156 (31.5)	169 (34.0)	
	55–64 *n* (%)	178 (35.9)	170 (34.3)	175 (35.3)	206 (41.5)	
	≥ 65 *n* (%)	96 (19.4)	78 (15.7)	75 (15.1)	66 (13.3)	
BMI (kg/m^2^)	<24.9 *n* (%)	161 (32.5)	145 (29.2)	137 (27.6)	134 (27.0)	0.3034
	25–29.9 *n* (%)	202 (40.7)	197 (39.7)	196 (39.5)	199 (40.0)	
	≥30 *n* (%)	133 (26.8)	154 (31.1)	163 (32.9)	164 (33.0)	

* Pearson’s chi-squared test; Q 1,2,3,4—quartiles 1,2,3,4; BMI—body mass index.

## Data Availability

Not applicable.

## Appendix A

**Table A1:** Characteristics of the study group. This table shows the number and percentage of the study participants in groups depending on age, place of residence, level of education, smoking status, and BMI.

Group		Total * *n* = 1985	Women * *n* = 1252	Men * *n* = 733
Age (years old)	<45	347 (17.5)	195 (15.6)	152 (20.7)
	45–54	594 (29.9)	398 (31.8)	196 (26.7)
	55–64	729 (36.7)	467 (37.3)	262 (35.7)
	≥65	315 (15.9)	192 (15.3)	123 (16.8)
Place of residence	Urban	1192 (60.1)	739 (59.0)	453 (61.8)
	Rural	793 (39.9)	513 (41.0)	280 (38.2)
Level of education	Non/primary	286 (14.4)	191 (15.3)	95 (13.0)
	Vocational	314 (15.8)	173 (13.8)	141 (19.2)
	Secondary	786 (39.6)	525 (41.9)	261 (35.6)
	Higher	599 (30.2)	363 (29.0)	236 (32.2)
Smoking status	Never	935 (47.1)	681 (54.4)	254 (34.7)
	Previous	630 (31.7)	328 (26.2)	302 (41.2)
	Current	420 (21.2)	243 (19.4)	177 (24.1)
BMI (kg/m^2^)	<24.9	577 (29.1)	419 (33.5)	158 (21.6)
	BMI 25–29.9	794 (40.0)	448 (35.8)	346 (47.2)
	BMI ≥ 30	614 (30.9)	385 (30.8)	229 (31.2)

* data are shown as number of participants (%); BMI—body mass index.
